# Construction and immune efficacy of recombinant pseudorabies virus expressing PrM-E proteins of Japanese encephalitis virus genotype І

**DOI:** 10.1186/s12985-015-0449-3

**Published:** 2015-12-10

**Authors:** Ping Qian, Xianwei Zhi, Bo Wang, Huawei Zhang, Huanchun Chen, Xiangmin Li

**Affiliations:** State Key Laboratory of Agricultural Microbiology, Huazhong Agricultural University, Wuhan, Hubei P.R. China; Laboratory of Animal Virology, College of Veterinary Medicine, Huazhong Agricultural University, Wuhan, Hubei P.R. China; Key Laboratory of development of veterinary diagnostic products, Ministry of Agriculture, Wuhan, 430070 P.R China

**Keywords:** Japanese encephalitis virus, Pseudorabies virus, Recombinant virus, PrM-E, Genotype I, Immune efficacy

## Abstract

**Background:**

Japanese encephalitis (JE) is an arboviral disease with high case fatality rates and neurologic or psychiatric sequelae among survivors in Asia, western Pacific countries and northern Australia. Japanese encephalitis virus (JEV) is the cause of JE and the emergence of genotype І (GI) JEV has displaced genotype III (GIII) as the dominant strains circulating in some Asian regions. The currently available JE vaccines are safe and effective in preventing this disease, but they are developed based on the GIII JEV strains.

**Methods:**

The recombinant virus PRV TK^−^/gE^−^/PrM-E^+^ which expressed the premembrane (prM) and envelope (E) proteins of JEV SX09S-01 strain (genotype I, GI) was constructed by homologous recombination between the genome of PRV TK^−^/gE^−^/LacZ^+^ digested with *Eco*RI and plasmid pIE-CAG-PrM-E-BGH. Expression of JEV PrM and E proteins was analyzed by Western blot analysis. Immune efficacy of PRV TK^−^/gE^−^/PrM-E^+^ was further evaluated in mouse model.

**Results:**

A recombinant pseudorabies virus (PRV TK^−^/gE^−^/PrM-E^+^) was successfully constructed. Mice experiments showed that PRV TK^−^/gE^−^/PrM-E^+^ could induce a high level of ELISA antibodies against PRV and JEV, as well as high titer of PRV neutralizing antibodies. After challenge with 1 × 10^7^ PFU virulent JEV SX09S-01 strain, the time of death was delayed and the survival rate was improved in PRV TK^−^/gE^−^/PrM-E^+^ vaccinated mice.

**Conclusions:**

PRV TK^−^/gE^−^/PrM-E^+^ is a potential vaccine candidate against PRV and JEV GI infection in the future.

## Background

Japanese encephalitis (JE) is a zoonotic disease and cause viral encephalitis with serious public health problem in Asia, western Pacific countries and northern Australia [1]. Japanese encephalitis virus (JEV), the etiological agent of JE, is the leading cause of epidemic encephalitis afflicting humans with 68,000 cases annually and 10–50 % fatality. 30–50 % of survivors result in permanent neuropsychiatric sequelae [2–6]. JEV is also an important pathogen in swine. It causes reproductive disorders with abortion and weak piglets [7]. Pigs and birds are the major amplifying hosts of JEV from which infected mosquitoes transmit the virus to humans [8].

JEV is an arbovirus with a single strand positive sense RNA belonging to the *flaviviridae* family and genus *flavivirus* [2, 3]. The JEV genome is about 11 kb in length with 5' and 3' non-translated regions (NTRs), coding three structural proteins (C, prM/M, E) and seven nonstructural proteins (NS1, NS2A, NS2B, NS3, NS4A, NS4B and NS5) [3, 9]. The envelope glycoprotein (E), composed of three domains, is the dominant immunogen capable of eliciting a high level of neutralizing antibodies. The membrane glycoprotein (PrM) is also a potent protein candidate for genetically engineered JEV vaccines [10–16].

Phylogenetic analyses indicate that JEV can be divided into five genotypes (GI-GV) based on the nucleotide sequence of E gene or the complete polyprotein gene [17–19]. GI-GV of JEV co-circulate in its geographically affected areas and GIII was previously dominant genotype [2]. However, GI strains have displaced GIII strains to become the predominant genotype in many Asian countries including Japan, China, Korea, Taiwan and Vietnam in recent years [20–27]. GI strains are considered to be more adapted to mosquitoes and pigs than to humans by achieving a replication cycle [28, 29]. The currently available JE vaccines are safe and effective in preventing this disease, but they are developed based on the GIII JEV strains [30]. There are two kinds of licensed JE vaccine for swine in China, both live-attenuated virus vaccine (SA14-14-2) and inactivated virus vaccine (HW1 stain) are derived from GIII viruses. Despite the sera from 12 to 18 month-old children vaccinated with licensed Japanese encephalitis chimeric virus (JE-CV) vaccine can neutralize recently isolated viruses, the live-attenuated JEV GIII vaccine is only partial protection for GI virus in swine [31–33]. Thus, new vaccines based on JEV G1 have been required for the prevention of pigs against virus infection.

In this study, a recombinant JEV vaccine was constructed by expression of the PrM-E proteins of JEV GI using an attenuated Pseudorabies virus vector (PRV TK^−^/gE^−^/LacZ^+^). The characters of the recombinant virus PRV TK^−^/gE^−^/PrM-E^+^ were evaluated and the protective immune responses to JEV were investigated in mouse model. Results showed that PRV TK^−^/gE^−^/PrM-E^+^ not only induce humoral immunity against JEV and PRV but also confer 80 % protection against 1 × 10^7^ PFU virulent JEV SX09S-01 strain challenge. PRV TK^−^/gE^−^/PrM-E^+^ is a promising candidate vaccine against JEV GI and PRV.

## Results

### Construction of the recombinant virus PRV TK^−^/gE^−^/PrM-E^+^

**Fig. 1:**
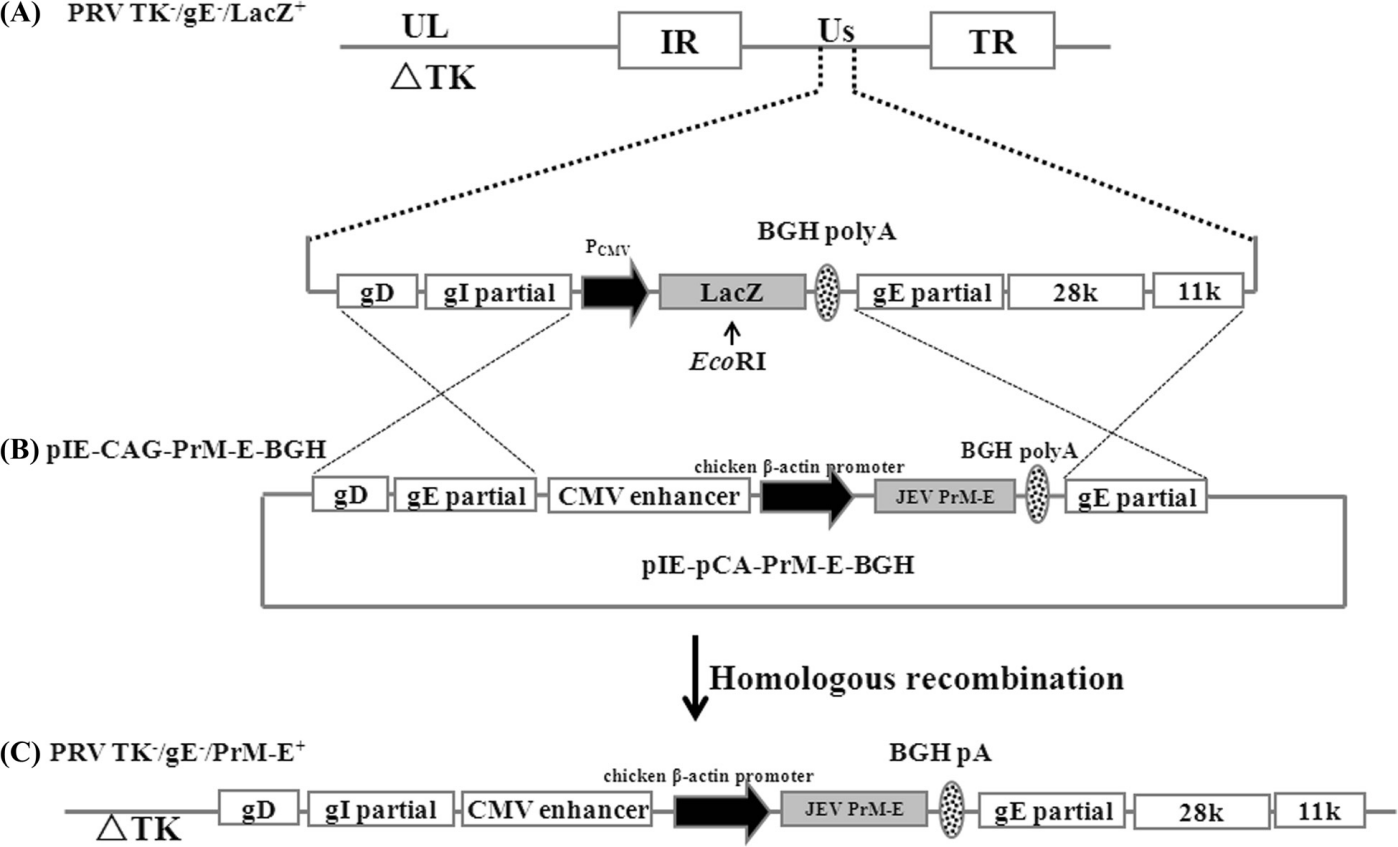
Schematic diagrams of the recombinant virus PRV TK^−^/gE^−^/PrM-E^+^ and transfer vactor. **a** The illustration of the genomic map of the parental attenuated PRV TK^−^/gE^−^/LacZ^+^ in which a LacZ expression cassette was inserted into the coding region of gE gene. The PRV genomic DNA is approximately 150 kb including a unique longe region (UL), a unique short region (Us), internal repeat (IR) and terminal repeat (TR). ∆TK represent the deleted thymidine kinase gene. **b** The map of transfer plasmid pIE-CAG-PrM-E-BGH in which partial coding region of gE and gI were replaced by a expression cassette containing the sequence of human cytomegalovirus (hCMV) enhancer, chicken β-actin promoter (CAG), PrM-E gene of JEV SX09S-01 strain, BHG poly-A signal (BHG pA). The PRV 28 k, 11 k, gE and gI partial and gD gene of PRV in the transfer plasmid to facilitate homologous. **c** Genome of recombinant PRV TK^−^/gE^−^/PrM-E^+^ virus, in which partial gI and gE gene are replaced by a PrM-E gene expression cassette. PRV TK^−^/gE^−^/PrM-E^+^ was constructed by homologous recombination on PK-15 cells between the genome of parental PRV TK^−^/gE^−^/LacZ^+^ and the transfer plasmid pIE-CAG-PrM-E-BGH

**Fig. 2:**
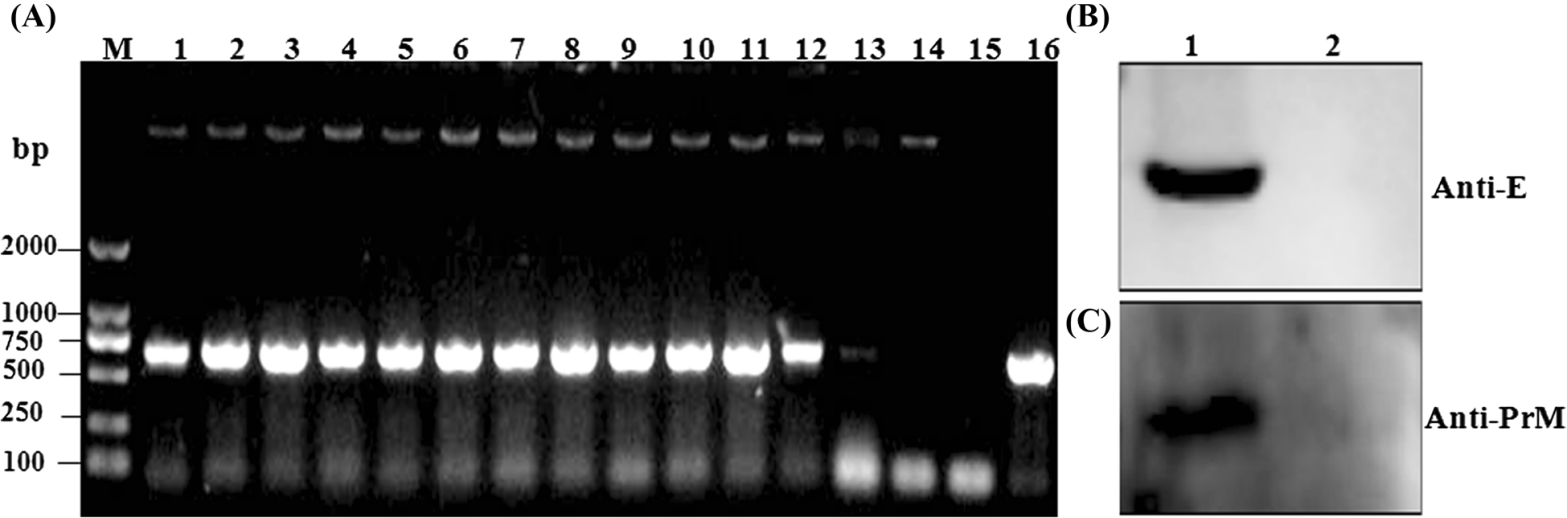
Identification of PRV TK^−^/gE^−^/PrM-E^+^ and expression of JEV PrM-E in PK-15 cells. **a** The total DNAs from virus-infected PK-15 cells were amplified by PCR with a pair of specific primers for JEV E gene according to JEV SX09S-01 strain and analyzed by electrophoresis. M: marker DL2000; 1 ~ 13: cells infected with different recombinant PRV clones; 14: cells infected with PRV TK^−^/gE^−^/LacZ^+^; 15: H_2_O; 16: positive control, plasmid pIE-CAG-PrM-E-BGH. **b & c** PK-15 cells infected with PRV TK^−^/gE^−^/PrM-E^+^ or parental virus PRV TK^−^/gE^−^/LacZ^+^ strain were collected for JEV E (B) and PrM (C) protein expression detection by western blotting. The proteins were detected in cells at 72 h post infection with the recombinant virus. Primary antibody is mAbs for E or for PrM and secondary antibody is goat anti-mouse IgG-HRP. Lane 1: cell lysates of PRV TK^−^/gE^−^/PrM-E^+^; Lane 3: cell lysates of PRV TK^−^/gE^−^/LacZ^+^

The recombinant virus PRV TK^−^/gE^−^/PrM-E^+^ was constructed by co-transfection with *Eco*RI-linearized genomic DNA of PRV TK^−^/gE^−^/LacZ^+^ strain (Fig. 1a) and transfer plasmid pIE-CAG-PrM-E-BGH (Fig. 1b) with an expression cassette containing JEV PrM-E gene which regulated by the immediate early gene promoter of human cytomegalovirus. After plaque purification by three rounds of plaque assay, the total DNAs from virus-infected PK-15 cells were amplified by PCR with specific primers (Table 1) for PrM-E gene and analyzed by electrophoresis. A specific 700 bp fragment containing PrM-E gene was detected from all recombinant plaques, but was absent in the control (Fig. 2a).

**Table 1:** Primers used in this study

Primer	Sequence 5' → 3'
JEF(Forward)^a^	TTG GAATTC ATG ATA AGA GGA GGG AAT G
JER(Reverse)^a^	TTT AGATCT TTA GGC ATG CAC ATT GGT CG
JEVeF^b^	GAT CTG TCG TAGCTCTTGGGTCACAG
JEVeR^b^	GCGTTGACACCCATCCAAAGAAG
IFN-γ-F^c^	TGGCATAGATGTGGAAGAA
IFN-γ-R^c^	GTGTGATTCAATCAATGACGCTTA
GAPDH-F^d^	GCCCAAGATGCCCTTCAGT
GAPDH-R^d^	CCTTCCGTGTTCCTACCCC

^a^Primers used to the clone the PrM-E gene; ^b^Primers used for the identification of recombinant virus PRV TK^−^/gE^−^/PrM-E^+^; ^c^The special primers for analysis of IFN-γ mRNA expression through real-time RT-PCR; d: Primers used for the control of real-time RT-PCR

To clarify the expression of PrM-E gene in the recombinant virus, PK-15 cells infected with recombinant virus PRV TK^−^/gE^−^/PrM-E^+^ or parental virus PRV TK^−^/gE^−^/LacZ^+^ strain were collected 72 h after infection. Cells were lysed for western blot analysis with mAbs for E or for PrM as first antibody. As shown in Fig. 2, specific protein bands, with molecular masses of approximately 53 kDa (Fig. 2b) and 19 kDa (Fig. 2c) corresponding to the expected size of E and PrM proteins, were appeared in the PRV TK^−^/gE^−^/PrM-E^+^ virus. But no specific proteins were detected in parental virus infected cells (Fig. 2b & c). Those results indicated that PrM and E proteins were correctly expressed in PRV TK^−^/gE^−^/PrM-E^+^ infected cells.

### Stability and growth properties of PRV TK^−^/gE^−^/PrM-E^+^

**Fig. 3:**
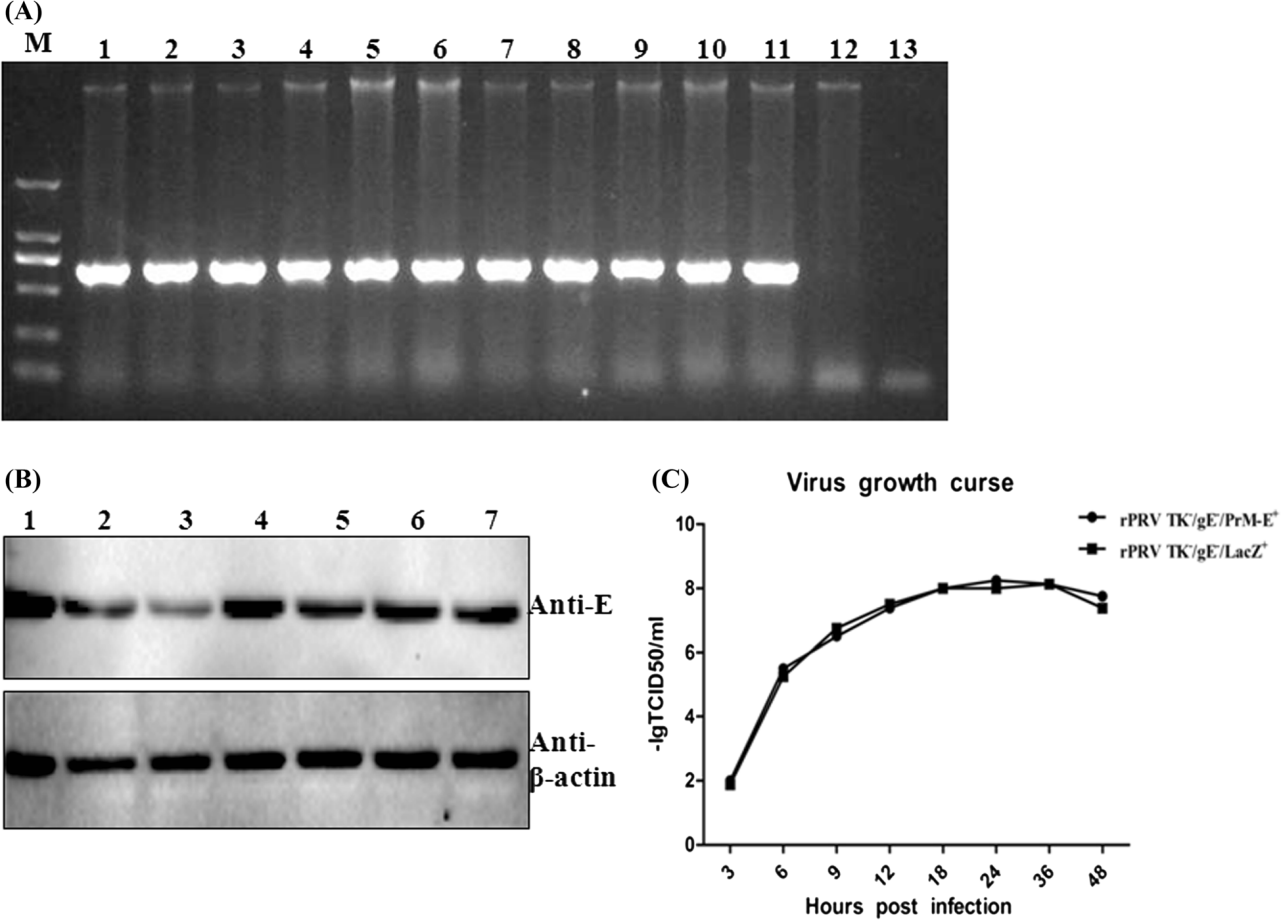
The biological characteristics analysis of PRV TK^−^/gE^−^/PrM-E^+^. **a** PRV TK^−^/gE^−^/PrM-E^+^ was grown on PK-15 cells sequentially for 20 passages and the total DNAs from virus-infected PK-15 cells were amplified by PCR with a specific primers for JEV E gene according to JEV SX09S-01 strain and analyzed by electrophoresis. M: marker DL2000; 1 ~ 11: cells infected with passages 11 to 20; 12: cells infected with PRV TK^−^/gE^−^/LacZ^+^; 13: H2O; **b** Genetic stability of protein identification for different generations by western blotting. PK-15 cells infected with different generations of PRV TK^−^/gE^−^/PrM-E^+^ were collected for JEV E protein expression detection by western blotting. 1 ~ 7: cells infected with passages 1, 2, 4, 8, 12, 16 and 20 of PRV TK^−^/gE^−^/PrM-E^+^. **c** Virus growth curves based on viral yield of supernatants harvested at different time points of PRV TK^−^/gE^−^/PrM-E^+^ or PRV TK^−^/gE^−^/LacZ^+^ infected PK-15 cells, and viral yield was measured by TCID_50_

To further assess the genetic stability and growth kinetics of PRV TK^−^/gE^−^/PrM-E^+^, the virus was grown on PK-15 cells sequentially for 20 passages. The viral DNA was extracted and analyzed after each passage using a pair of E-specific primers by PCR (Table 1). The expression of E protein was also determined by western blot analysis (Fig. 3). Results showed that the E gene was stably inserted into the PRV genome (Fig. 3a) and robustly expressed in the recombinant PRV TK^−^/gE^−^/PrM-E^+^ infected cells (Fig. 3b).

To compare the growth of PRV TK^−^/gE^−^/PrM-E^+^ and PRV TK^−^/gE^−^/LacZ^+^, one-step growth curve was conducted to address whether the insertion of PrM-E gene fragment affected the replication of PRV. The 50 % tissue culture infective dose (TCID_50_) of recombinant virus was similar to parental virus at 0, 3, 6, 12, 18, 24, and 30 h post-infection (Fig. 3c). These data indicated that insertion of exogenous genes in the gE locus did not affect replication of PRV.

### JEV-specific immune responses in mice

**Fig. 4:**
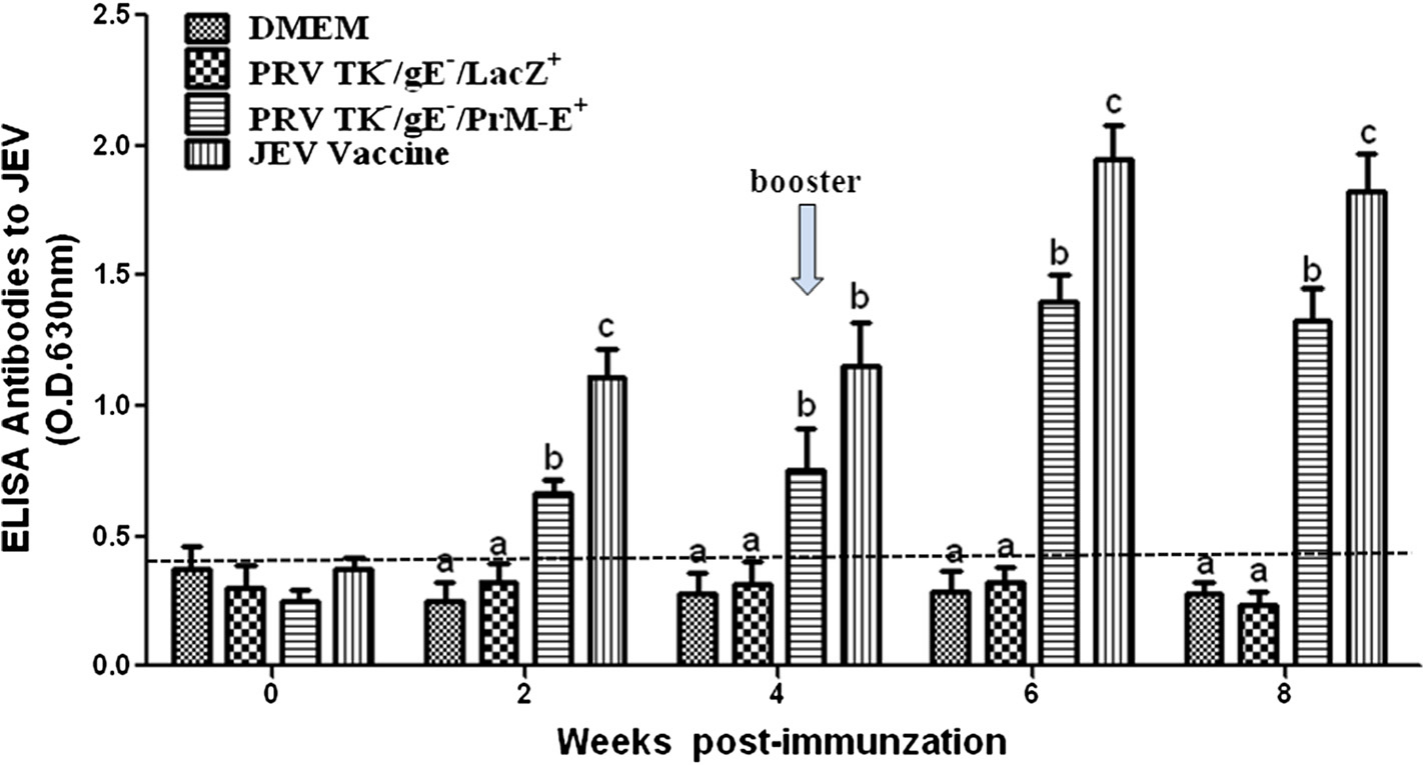
Humoral immune responses of JEV in immunized mice. Serum samples were collected at weekly intervals after immunization to detect the presence of the JEV-specific antibodies by indirect ELISA. JEV-specific antibodies were found in serum samples at different times. Data represent the mean ± SEM. Different letters indicate a statistically significant difference between the different experimental groups (*P* < 0.05). Different letters (a, b, c) indicate a statistically significant difference between the different experimental groups (*P* < 0.05)

To determine whether recombinant virus PRV TK^−^/gE^−^/PrM-E^+^ could induce JEV-specific immune responses, serum samples of PRV TK^−^/gE^−^/PrM-E^+^, PRV TK^−^/gE^−^/LacZ^+^, DMEM and JEV inactivated vaccine vaccinated mice were collected and determined using an indirect ELISA at different weeks. JEV-specific antibodies were first detected at 2 week post-immunization (wpi) and PRV TK^−^/gE^−^/PrM-E^+^ group developed significantly higher JEV-specific ELISA antibody titers than PRV TK^−^/gE^−^/LacZ^+^ and DMEM groups (*P* < 0.05). At 6 wpi, the JEV antibodies reached a peak after booster immunization in the PRV TK^−^/gE^−^/PrM-E^+^ and JEV inactivated vaccine groups (Fig. 4). The inactivated vaccine group developed significantly higher JEV-specific ELISA antibody titers than the other three groups at 2 and 6 wpi (*P* < 0.01), but there was no difference between PRV TK^−^/gE^−^/PrM-E^+^ and JEV inactivated vaccine groups at 4 and 8 wpi (*P* > 0.05).

The ability of the serum samples to neutralize JEV was evaluated using plaque reduction neutralization test (PRNT) assay. Mice vaccinated with PRV TK^−^/gE^−^/PrM-E^+^ had a little of JEV neutralizing antibody titers. Sera diluted with 1:4 and 1:8 have the ability to neutralize 200PFU JEV and reduce the plaque number. The plaque number is about 64.5 ± 0.5 in the control virus without serum. While the plaque number of virus mixed with different dilution of sera inoculated with PRV TK^−^/gE^−^/PrM-E^+^ is 23.5 ± 1.5 (1:4 dilution), 35.0 ± 1.0 (1:8 dilution), 55.5 ± 0.5 (1:16 dilution), 60.5 ± 1.5(1: 32 dilution), respectively (Table 2). Mice vaccinated with JEV inactivated vaccine had higher neutralizing antibody titers than PRV TK^−^/gE^−^/PrM-E^+^ group. The plaque number of virus mixed with sera inoculated with JEV inactivated vaccine is 11.0 ± 1.0 (1:4 dilution) and 34.0 ± 1.5 (1: 32 dilution), respectively (Table 2). The PRV TK^−^/gE^−^/LacZ^+^ group did not exhibit any neutralizing antibody against JEV throughout the experiment. All results showed that the neutralizing antibody titers were not high in the PRV TK^−^/gE^−^/PrM-E^+^ group.

**Table 2:** The plaque numbers of serum samples in immunized mice by plaque reduction neutralization test (PRNT) assay

Dilution of Serum^a^	Numbers of Plaque by PRNT			
	DMEM	PRV TK^−^/gE^−^/LacZ^+^	PRV TK^−^/gE^−^/PrM-E^+^	JEV Vaccine
1:4	64.5 ± 0.5	59.5 ± 1.5	23.5 ± 1.5	11.0 ± 1.0
1:8	62.5 ± 2.5	57.5 ± 2.5	35.0 ± 1.0	19.5 ± 0.5
1:16	63.5 ± 1.5	60.5 ± 0.5	55.5 ± 0.5	24.0 ± 1.0
1:32	62.5 ± 0.5	63.5 ± 1.5	60.5 ± 1.5	34.0 ± 1.5
1:64	63.0 ± 1.5	62.5 ± 0.5	63.5 ± 0.5	62.5 ± 2.5

^a^The sera of mice at 8 weeks post immunization was diluted and mixted with 200PFU of JEV SX/09/01 strain for 1 h at 37 °C, and then added to BHK-21 in 24-well plate by PRNT assay

**Fig. 5:**
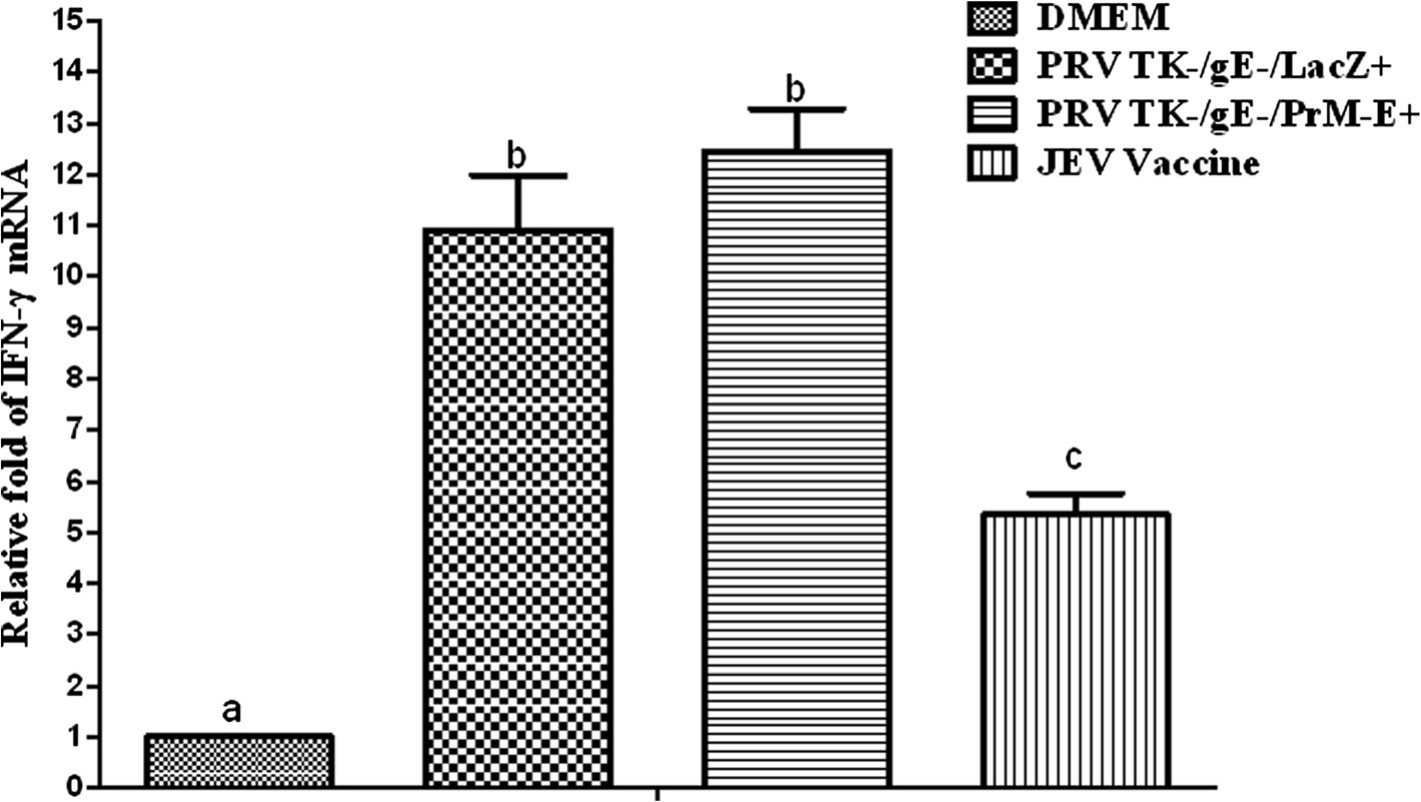
Relative IFN-γ gene expression in mice. The level of IFN-γ per group in serum samples of mice. Data represent the mean ± SEM. Different letters indicate a statistically significant difference between the different experimental groups (*P* < 0.05). Different letters (a, b, c) indicate a statistically significant difference between the different experimental groups (*P* < 0.05)

To evaluate cellular immune response to JEV, splenocytes from immunized mice were stimulated with UV-inactivated JEV. The IFN-γ mRNA expression in lymphocytes was analyzed by real-time RT-PCR. As shown in Fig. 5, PRV TK^−^/gE^−^/PrM-E^+^ and PRV TK^−^/gE^−^/LacZ^+^ groups elicited higher IFN-γ mRNA expression than JEV inactivated vaccine group (*P* < 0.05). Statistical analyses indicated that there were no significant differences between PRV TK^−^/gE^−^/PrM-E^+^ and PRV TK^−^/gE^−^/LacZ^+^ group (*P* > 0.05).

### PRV-specific humoral immune responses in mice

**Fig. 6:**
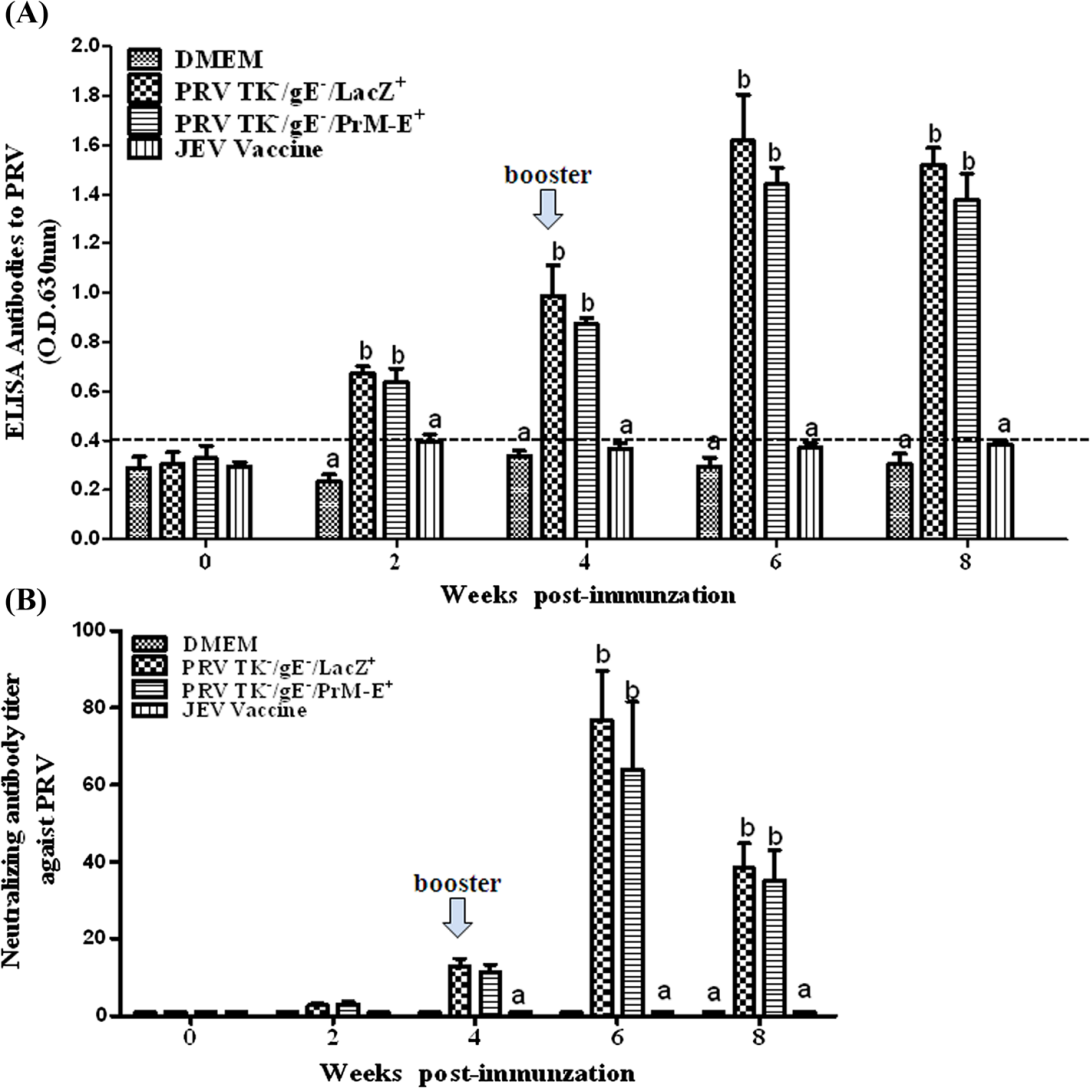
Humoral immune responses against PRV in immunized mice. Serum samples were collected at weekly intervals after immunization. **a** Serum samples of PRV-specific antibodies using the PRV-gB Antibody Test Kit by indirect ELISA, according to the manufacturer’s instructions. **b** PRV Neutralizing antibodies were measured in serum samples at different times by serum neutralization test. PRV Neutralizing antibodies were calculated and expressed as the reciprocal of the highest serum dilution that inhibits 50 % of the culture wells of the PRV replication. Data represent the mean ± SEM. Statistical significance was assessed by Student’s *t*-test (***: *P* < 0.001). Data represent the mean ± SEM. Different letters (a, b, c) indicate a statistically significant difference between the different experimental groups (*P* < 0.05)

To analyse whether PRV TK^−^/gE^−^/PrM-E^+^ could induce PRV-specific immune responses after immunization, we determined the specific antibodies in the vaccinated mice by indirect ELISA. As shown in Fig. 6a, all animals vaccinated with PRV TK^−^/gE^−^/PrM-E^+^ and PRV TK^−^/gE^−^/LacZ^+^ were positive at 4 wpi. The mean titers of PRV specific antibodies significantly increased in the mice vaccinated with PRV TK^−^/gE^−^/PrM-E^+^ and PRV TK^−^/gE^−^/LacZ^+^ and were higher than those in the mice vaccinated with DMEM and JEV inactivated vaccine (*P* < 0.01). By contrast, the PRV specific antibodies were not detected in the sera of DMEM and JEV inactivated vaccine.

Moreover, a serum neutralization test was also performed to determine the antibody against PRV. At 4 wpi, low to no neutralization antibodies against PRV was detected in PRV TK^−^/gE^−^/PrM-E^+^ and PRV TK^−^/gE^−^/LacZ^+^ groups. However, both recombinant virus and parental virus groups elicited high neutralization antibody titers at 6 wpi, there was no difference between the two groups (*P* > 0.05) (Fig. 6b). No neutralizing antibodies were detected in DMEM and JEV inactivated vaccine groups. These results showed that PRV TK^−^/gE^−^/PrM-E^+^ induced a high level of PRV ELISA and neutralizing antibodies.

### Vaccine Efficacy against JEV Challenge in Mice

Animal experiments were conducted to evaluate the protective efficacy of the recombinant virus PRV TK^−^/gE^−^/PrM-E^+^ against JEV challenge. Mice (ten per group) were intraperitoneally inoculated with 1 × 10^7^ PFU of JEV SX09S-01 strain at 8 weeks after the primary immunization and checked daily for survival. The characteristic signs of JE such as hunched posture, ruffled fur, depressions, tremors and hind-leg paralysis were appeared at 5 day after infection (dpi) in the mice of DMEM group. 8 mice vaccinated with the recombinant virus PRV TK^−^/gE^−^/PrM-E^+^ survived without showing characteristic signs of JE. Mice vaccinated with JEV inactivated vaccine showed 100 % survival rates. However, survival rates of 10 % were observed in mice vaccinated with PRV TK^−^/gE^−^/LacZ^+^. These results indicated that immunization with PRV TK^−^/gE^−^/PrM-E^+^ could confer 80 % protective efficacy against the lethal challenge in mice.

## Discussion

Japanese encephalitis (JE) is a serious arthropod-borne disease in southern and eastern Asia [1] and JEV can cause epidemic viral encephalitis in humans and reproductive disorders in swine. Currently JEV genotype I (GI) strains have displaced genotype III (GIII) strains to become the predominant genotype in some Asian countries including China and Taiwan [23, 25]. However, inactivated JEV GIII vaccine can only reduce strain-specific neutralizing antibody titers and partial protection against G1 virus in mice and swine [32–35]. Swine is an important amplifying and overwintering host for JEV and plays a crucial role in the human encephalitis epidemics [8]. The prevention and control of JEV infection in swine are very important to the prevention of humans. In view of the genotype shift, there is an urgent need to develop new JEV vaccines for swine to control this epidemic.

Pseudorabies virus (PRV) infection causes severe financial losses in the animal husbandry [36]. Our and other laboratories have confirmed that live attenuated pseudorabies vaccines could stimulate strong cell-mediated immune responses and high titers of neutralizing antibody against PRV and were successfully used to control PRV infection [37, 38]. Meanwhile, live attenuated PRV have been used as a highly economical promising candidate vector for the development of bivalent vaccine from animal viruses [39, 40]. *Xu et al.* had determined that PRV TK^−^/gE^−^/NS1^+^ expressed NS1 gene of JEV (SA14-14-2) could induce JEV-specific humoral and cellular immune responses [41].

In this study, we have successfully constructed a recombinant virus PRV TK^−^/gE^−^/PrM-E^+^ expressing the premembrane (prM) and envelope (E) proteins of JEV SX09S-01 strain (GI). Precursor protein PrM-E can be accurately cleaved into PrM and E proteins by host signal peptidase (Fig. 2a and b). It has been confirmed that prM and E genes had been stably inserted and expressed, which did not affect the infection and replication of PRV (Fig. 3c). We further investigated the efficacy of the recombinant virus PRV TK^−^/gE^−^/PrM-E^+^ against JEV. Results indicated that both recombinant virus PRV TK^−^/gE^−^/PrM-E^+^ and parental virus induced high-neutralizing and ELISA antibodies against PRV and there are not significant differences between them (Fig. 6). Immunization of PRV TK^−^/gE^−^/PrM-E^+^ could induce humoral and cellular immune responses (Figs. 4 and 5) in the mouse model.

Specific JEV-antibodies were detected in the mice by indirect ELISA assay. These results showed that PRV TK^−^/gE^−^/PrM-E^+^ and JEV inactivated vaccine could induce high levels of the JEV-specific antibodies compared with the negative controls, PRV TK^−^/gE^−^/LacZ^+^ and DMEM (Fig. 4). This is consistent with the previous study, which showing the recombinant E protein could induce a higher titer of IgG1 indicating Th2-cells response [42, 43]. The ability of the serum samples to neutralize JEV SX09S-01 strain was also evaluated using plaque reduction neutralization test (PRNT) assay at 8 wpi. But the neutralizing antibody titers were not high in the PRV TK^−^/gE^−^/PrM-E^+^ group, only the sera with 1:4 and 1:8 dilution showed special neutralizing effect (Table 2).

The cellular immune responses were also assessed by evaluating the mRNA expression levels of IFN-γ which induced by the Th1 cellular response, the high level of IFN-γ expression were induced in mice immunized with PRV TK^−^/gE^−^/PrM-E^+^ and PRV TK^−^/gE^−^/LacZ^+^. These results showed that the live-attenuated PRV could stimulate good cellular immunity (Fig. 5). Although JEV inactivated vaccine could elicit higher humoral immune responses than that of PRV TK^−^/gE^−^/PrM-E^+^, the cellular immune responses are lower compared to PRV TK^−^/gE^−^/PrM-E^+^. The results of JEV challenge showed that mice inoculated with inactivated JEV vaccine were completely protected and PRV TK^−^/gE^−^/PrM-E^+^ conferred 80 % protection (Fig. 7). In addition, DMEM control group showed typical JE signs and PRV TK^−^/gE^−^/LacZ^+^ conferred 10 % protection and it showed that cellular immunity play a crucial role against JEV challenge [44].

## Conclusion

In summary, the recombinant PRV TK^−^/gE^−^/PrM-E^+^ was successfully constructed and it could efficiently express the glycoprotein PrM-E and induced humoral and cellular immunity against JEV. 80 % mice were protected from challenge with JEV. Thus, PRV TK^−^/gE^−^/PrM-E^+^ may serve as a candidate for generating a novel vaccine that can be used for controlling PRV and JEV GI strain infection. Certainly, additional studies will be conducted to evaluate the immunogenic and protective effects of these vaccines in pigs. To enhance the immune response, we are constructing recombinant viruses by co-expressing immunomodulatory molecules, such as GMCSF [45].

## Methods

### Viruses, cells and inactivated vaccine

The live-attenuated PRV vaccine strain (PRV TK^−^/gE^−^/lacZ^+^) was previously constructed and propagated on PK-15 cells used as a parental virus vector in our laboratory [46]. Japanese encephalitis virus (JEV) SX09S-01 strain, genotype GI strain, were propagated on BHK-21 cells and stored at −80 °C to be used for animal challenge [23]. Pig kidney cells (PK-15, ATCC) and Baby hamster kidney-21 cells (BHK-21, ATCC) were grown and maintained in Dulbecco’s modified Eagle’s medium (DMEM, Gibco, USA) supplemented with 10 % heat-inactivated fetal bovine serum (FBS; Gibco, New Zealand) at 37 °C with 5 % CO_2_. JEV-inactivated vaccine was purchased from Jingmu Vet-biological Products Company (Wuhan, China).

### Plasmids construction

All primers and the sequences used in this study were listed in Table 1. The PrM-E gene fragment was cloned from JEV SX09S-01 strain with a pair of primers JEF (Forward) and JEF (Reverse). The fragment was cloned into the *Eco*RI/*Bgl*II sites of the vector pCAGGS (invitrogen) under the control of the chicken β-actin promoter to generate pCA-PrM-E. A PrM-E gene expression cassette containing the enhancer of human cytomegalovirus (hCMV), chicken β-actin promoter (CAG) and PrM-E gene from pCA-PrM-E was further subcloned into the *Sal*I-*Bgl*II sites of universal transfer vector pIE-CMV-BGH-CMV-SV40 (constructed previously in our laboratory) and the resulting transfer plasmid was named as pIE-CAG-PrM-E-BGH (Fig. 1b).

### Construction of recombinant viruses PRV TK^−^/gE^−^/PrM-E^+^

The recombinant virus PRV TK^−^/gE^−^/PrM-E^+^ was constructed by homologous recombination between the genome of PRV TK^−^/gE^−^/LacZ^+^ digested with *Eco*RI and plasmid pIE-CAG-PrM-E-BGH (Fig. 1) according to the method described by Qian P [39]. Co-transfection was conducted in PK-15 cells using Lipofectamine 2000 (Invitrogen) with the method of manufacturer’s instruction. After cytopathogenic effect (CPE) appeared, the transfected cells were collected 48 ~ 72 h later. The recombinant virus PRV TK^−^/gE^−^/PrM-E^+^ was subjected to plaque purification and identification with PCR for amplifying a small fragment of PrM-E gene with a pair of primers JEVeF and JEVeR (Table 1). Expression of JEV PrM and E proteins was analyzed by Western blot analysis.

### Detection the expression of PrM and E protein

PrM and E protein expression was evaluated by Western blot analysis using monoclonal antibodies (MAbs). The mAbs against E and PrM were kindly provided by professor Cao shengbo and Doctor Song yunfeng (Huazhong Agriculture University) [47]. The lysates from PRV TK^−^/gE^−^/PrM-E^+^ infected cells were separated by SDS-PAGE gel and transferred to polyvinylidene difluoride membranes (Roche). The nonspecific antibody-binding sites were blocked by 5 % skim milk in Tris Buffered Saline (TBS). The E and PrM MAbs and horseradish peroxides (HRP)-conjugated goat anti-mouse IgG (1:2000 dilution, ABclonal, Wuhan, Hubei, China) were used as the primary and the secondary antibody. The bands were visualized using Electro-Chemi-Luminescence kit (Thermo, USA) according to the manufacturer’s instructions.

### Stability and Growth properties of PRV TK^−^/gE^−^/PrM-E^+^

To analyze the genetic stability of the foreign gene in the recombinant virus PRV TK^−^/gE^−^/PrM-E^+^, the virus was sequentially grown on PK-15 cells and viral DNA was extracted and analyzed after each passage using E-specific primers by PCR. At the same time, the expression of JEV E protein after each passage was determined by Western blot analysis. One step growth kinetic assay was performed to determine the titer of recombinant virus PRV TK^−^/gE^−^/PrM-E^+^ as previously described [48]. Briefly, cells were infected at a multiplicity of infection (MOI) of 0.02 and harvested at different time points. The virus was titrated by 50 % tissue culture infective dose (TCID_50_) assay in PK-15 cells.

### Immunization and viral challenge of mice

Four-week old female SPF Balb/c mice were purchased from Zhongnan Hospital of Wuhan University (Hubei Province, China) and randomly divided into four groups (10 mice per group). Groups A and B were inoculated intramuscularly (i.m.) with 100 μl (1.0 × 10^6^ TCID_50_) of PRV TK^−^/gE^−^/PrM-E^+^ and PRV TK^−^/gE^−^/lacZ^+^, respectively. Group C and D were inoculated i.m. with 100 μl of DMEM and 100 μl JEV inactivated vaccine (SA14-14-2, immunization dose reference to instructions) as controls. Booster injection was administered with the same dose at 4 weeks post-immunization (wpi). At 8 weeks after the primary immunization, mice were intraperitoneally inoculated with 100 μl (1 × 10^7^ PFU) of JEV SX09S-01 strain and checked for survival [49]. After JEV challenge, the signs of JE and survival rates were monitored daily for 10 days as previously described [44].

### Antibody detection using ELISA assays

Serum samples were collected at 0, 2, 4, 6 and 8 weeks after the initial immunization and were tested using an indirect ELISA with PRV or JEV antibody ELISA kit (Wuhan KeQian Biological Co., Ltd.), which was placed on microplates coated with gB protein of PRV or inactivated JEV virus antigen, according to the manufacturer’s instructions. The sera were dealed with 1:40 dilution and the samples were considered positive when OD_630_ > 0.40 while negative when OD_630_ < 0.40 for PRV, and positive when OD_630_ > 0.45 while negative when OD_630_ < 0.45 for JEV.

### Serum neutralization test

PRV neutralizing antibodies were measured in serum samples at different times. The serum neutralization test was performed as previously described [39]. 50 μl of serum samples were two-fold serially diluted and mixed with equal volume of viral suspension containing 100 TCID_50_ PRV Ea strain for 1 h at 37 °C in 96-well flat-bottomed tissue culture plates (Nunc, USA). The mixture was then inoculated for 1 h at 37 °C and 5 % CO_2_.100 μl of PK-15 cells suspension (ca.1.0 × 10^6^ ml^−1^) was added to each well and inoculated for another 4–5 days. The plates were observed under a microscope for cytopathic effect.

The JEV neutralizing antibodies were also detected with plaque reduction neutralization test (PRNT) by using monolayers of BHK-21 cells according to the method of Yang DK [43]. Briefly, 3 × 10^4^ BHK-21 cells were seeded into 24-well plates and incubated until a monolayer formed at 37 °C. The heat inactivated sera were two-fold serially diluted and mixed with an equal volume of 200 PFU of JEV SX09S-01 strain. The mixture were incubated for 1 h at 37 °C and then added to BHK-21 cells monolayer in 24-well plate. Then the virus solution was aspirated, cells in each well were washed three times with phosphate-buffered saline (PBS, pH 7.4) and overlaid with 1.0 ml of 4 % carboxymethylcellulose (CMC) in growth medium (DMEM without phenol red, supplemented with 3 % FCS) and incubated at 37 °C and 5 % CO_2_ for 5 days. After virus plaques were formed, cells were fixed with 10 % formalin and stained with 1 % crystal violet. The PRNT_50_ titers were calculated as the reciprocal of the dilution of serum that reduced the plaque number by at least 50 % of that in the virus control.

### Analysis of IFN-γ mRNA expression through real-time RT-PCR

Splenocytes from mice immunized were isolated from spleen and were cultured in 12-well plates for 20 h with 20 μL of UV-inactivated JEV (SX09S-01 strain, MOI = 1). Total RNA was extracted and the cDNA product was further amplified with SYBR® Green Real-time PCR Master Mix (ToYoBo) and specific primers (Table 1). Each experiment was performed in triplicate. PCR amplification was performed under the following conditions: 2 min at 50 °C, 10 min at 94 °C, and 40 cycles of 15 s at 94 °C and 1 min at 5 °C. Gene expression was determined using the relative quantity and then analyzed as previously described [44].

### Statistical analysis

Statistical analysis was conducted using the GraphPad Prism Version 5 (GraphPad Software, La Jolla, CA, USA, 2012). One-way ANOVA was used for statistical analyses among different groups. *P*-value less than 0.05 was considered statistically significant.
